# A Social Force Evacuation Model with Guides Based on Fuzzy Clustering and a Two-Layer Fuzzy Inference

**DOI:** 10.1155/2022/7700511

**Published:** 2022-10-14

**Authors:** Qian Xiao, Jiayang Li

**Affiliations:** ^1^School of Intelligent Science and Engineering Shenyang University, Shenyang, Liaoning 110044, China; ^2^School of Business Administration Northeastern University, Shenyang, Liaoning 110000, China

## Abstract

Current emergency management research mainly specifies the positions of evacuation guides from a knowledge base of experience, disregarding the subjective perceived decision-making of pedestrians caught in an emergency situation. Therefore, in this paper, a fuzzy inference system for pedestrians to select guides is designed from the perspective of pedestrians, and a crowd evacuation model with guides under limited vision is constructed. First, selecting the indoor evacuation of people with limited vision as the context, the number and optimal initial positions of guides are determined by a Gaussian fuzzy clustering algorithm. Next, a two-layer fuzzy inference system based on a multifactor pedestrian selection guide is established. Then, from the comprehensive perspective of managers and pedestrians, an improved social force evacuation model with guides is constructed. A comparison of the evacuation times and evacuation processes of known methods with different scene population distributions is analyzed through simulations. The results show that the guide setting scheme of the improved model is more conducive to reducing evacuation times and balancing exit utilizations. The model can provide a basis for emergency management decision-making departments to formulate more flexible guidance strategies.

## 1. Introduction

During an emergency, an important safety measure is to quickly release a dense crowd from a crowded space. A guidance strategy can effectively control the extreme emotions of the crowd during the evacuation process. Therefore, it is necessary to scientifically and reasonably evacuate and guide the crowd when a crisis occurs [1–3]. Previous studies have shown that [4–7] sending trained guides to organize and lead the crowd to find exits has a practical benefit in alleviating the crowd's tension, reducing secondary injuries, and improving the crowd evacuation efficiency.

Some researchers have analyzed the influence of facilitators [8, 9]. The research results prove that the instructions of the guide are particularly important. These conclusions provide an important premise for the formulation and implementation of an evacuation guide strategy. Researchers have conducted in-depth research on the guide setting strategy. Wang et al. [10] proposed an extended evacuation field model and analyzed the effective location and an optimal number of evacuation guides through simulation experiments. Wu and Sun [11] developed a hybrid model based on social forces and agents to evaluate the guide setting of large-scale groups. Hou et al. [12] explored the influence of the number and location of evacuation guides on the dynamic process of crowd evacuation, and proposed that the optimal scheme is to establish an evacuation guide for the same number of exits in the center of a multiexit room.

Considering the differences between evacuation scenes and evacuees, researchers began to analyze the optimal setting strategy of evacuation guides from a behavioral decision-making perspective. Ma et al. [5] discovered that the optimal number of evacuation guides is related to the site environment and crowd size. Cao et al. [13] discussed the impact of evacuation guides on crowd evacuation efficiency in four scenarios: a random distribution, central distribution, uniform distribution, and exit distribution. This study is helpful for developing effective evacuation management strategies in emergency situations. Through simulations based on an extended social force model, Gerakakis et al. [14] concluded that the effects of guides on pedestrian evacuations with limited visibility are dual and related to the neighbor density within the visual field. Considering the advantages of the fuzzy logic method in handling uncertain fuzzy information, some scholars have applied fuzzy theory to the study of crowd evacuation. Yang et al. [15] proposed a crowd evacuation model based on fuzzy logic and the selection of followers to guide. The simulation data verified the feasibility of the proposed selection method. Gerakakis et al. [16] proposed the incorporation of fuzzy logic principles in a cellular automata model that simulates crowd dynamics and crowd evacuation processes, with the usage of a Mamdani-type fuzzy inference system, resulting in a realistic and rather efficient modeling approach. Al-Ahmadi et al. [17] developed an urban cellular automata model that used fuzzy set theory to capture the uncertainty associated with transition rules. Fu et al. [18] proposed a discrete evacuation model defined on the cellular space according to fuzzy theory, which can describe the imprecise and subjective information in the process of pedestrian evacuation. Villanueva et al. [19] designed a fire evacuation system (FES) using Mamdani-type fuzzy logic control to improve the responsiveness and reliability of fire detection.

In summary, establishing a reasonable evacuation guide is an effective way to change pedestrian behavior through a guidance strategy, and it is at the forefront of current evacuation strategy research. At present, researchers have obtained some constructive conclusions about the setting of evacuation guides from the perspectives of the site area, crowd size, and visual field conditions. However, the location of the guide is specified from the experiments or perspective in some studies. Moreover, they assumed that pedestrians fully abide by the assigned guide, disregarding the subjective choice behavior of pedestrians. Some research studies proposed to apply fuzzy theory to crowd evacuation to describe the uncertain behavior of pedestrians, but failed to comprehensively consider the pedestrians' location distribution and their perceived decision-making abilities. Therefore, in this paper, the fuzzy theory is used to establish a setting scheme and pedestrian decision inference system; the exit selection mechanism of guides and the movement mechanism of pedestrians with social force are improved; and a new social force evacuation model involving the guide is constructed.

Compared with K-means and other “hard clustering” algorithms [20–22], the Gaussian fuzzy clustering belongs to the “soft clustering” methods. The Gaussian fuzzy clustering is more flexible [23–25] and can determine the clustering members according to the probability of obtaining a better clustering effect. Therefore, this paper proposes a guide selection method based on the Gaussian fuzzy clustering and constructs a new guide participation social force evacuation model based on fuzzy theory. First, the model adopts the Gaussian fuzzy clustering method to determine the optimal number and location of guides according to the crowd distribution. Second, a multifactor fuzzy reasoning system is constructed to reflect pedestrians' independent choice decisions when facing multiple guides so that the individual behavior in the improved model has more intelligent characteristics. The remainder of this paper is structured as follows: in the third section, a crowd evacuation model based on improved social force is established by combining the setting of guides and the selection decisions of pedestrians. In the fourth section, the effectiveness of the improved model is verified, and the influence of different guide settings on the evacuation efficiency is discussed. Concluding remarks and future research directions are then discussed.

## 2. Guide Setting Algorithm Based on Gaussian Fuzzy Clustering

### 2.1. Basic Theory of the Gaussian Mixture Model

The basic principle of the Gaussian mixture model (GMM) is that multiple Gaussian distribution functions are linearly combined into an approximate overall Gaussian distribution function [26], and the calculation result is that the sample data points belong to a Gaussian component with probability. It is supposed that there is a dataset *X*={*x*_1_, *x*_2_, *x*_3_,…, *x*_*n*_} with *n* samples, where *x*_*j*_ is a *d*-dimensional random variable representing a sampling point, that is, *x*_*j*_=(*x*_*j*_^1^, *x*_*j*_^2^, *x*_*j*_^3^ … *x*_*j*_^*d*^)^*T*^. The Gaussian mixture model has *K* Gaussian components. Next, *x*_*j*_ belongs to the probability density function *p*_*k*_(*x*_*j*_|*μ*_*k*_, Σ_*k*_) of the *k*-th Gaussian component, as shown in equation (1). The probability density function *P*(*x*_*j*_|Θ) approximating the overall distribution is obtained by linearly weighting the Gaussian component, as shown in

(1) $$\begin{array}{c} p_{k}\left(x_{j}\left|\mu_{k}\right.,\Sigma_{k}\right)={\left(2\pi \right)}^{-\left(d/2\right)}{\left|\Sigma_{k}\right|}^{-\left(1/2\right)}\cdot \exp\;\left[-\frac{1}{2}{\left(x_{j}-\mu_{k}\right)}^{T}\Sigma_{k}^{-1}\left(x_{j}-\mu_{k}\right)\right]\text{,} \end{array}$$

(2) $$\begin{array}{c} P\left(x_{j}\left|\Theta \right.\right)=\overset{K}{\underset{k=1}{\sum }}\omega_{k}\cdot p_{k}\left(x_{j}\left|\mu_{k}\right.,\sigma_{k}\right)\text{,} \end{array}$$

where Θ is the parameter vector set of *K* Gaussian components, expressed as Θ={*θ*_1_, *θ*_2_, *θ*_3_ … *θ*_*K*_}; *θ*_*k*_ is the parameter set *θ*_*k*_={*ω*_*k*_, *μ*_*k*_, Σ_*k*_} of the *k*-th Gaussian component, which is composed of the weight *ω*_*k*_, mean *μ*_*k*_, and covariance Σ_*k*_ of the Gaussian component.

When the Gaussian mixture model clusters the dataset, it is impossible to determine to which Gaussian component each data point belongs. Therefore, the expectation maximization (EM) algorithm is generally utilized to estimate the parameters of the Gaussian mixture model [27]. The EM algorithm is an effective iterative process. Step *E* calculates the posterior probability of hidden variables according to the parameters of the previous iteration, which is the conditional expected value of the hidden variables. Step *M* maximizes the likelihood function to obtain a new parameter value.

### 2.2. Guide Setting Algorithm

The output of the Gaussian mixture model is a series of probabilities. The Gaussian component with the largest probability value is selected as the data attribution category. This method is applied to calculate the initial position and number of guides during the process of evacuation. It is supposed that there are *n* pedestrians in an indoor site in case of an emergency and that the pedestrian dataset is PER={per_*j*_}, *j*=1,2,…, *n*, where per_*j*_ is the location data of the *j*-th pedestrian. It is supposed that the management sends *K* guides to the site to lead the crowd to evacuate, which constitutes the component of the GMM, in which the Gaussian component parameter set of the *k*-th (*k*=1,2,…, *K*) guide *g*_*k*_ is expressed as *θ*_*k*_={*ω*_*k*_, *μ*_*k*_, Σ_*k*_}. Next, using the Gaussian mixture model to determine the number and initial positions of guides is the process of determining the best Gaussian fuzzy clustering parameter set. The algorithm is expressed as follows:

Step 1: Input the position coordinate set PER={per_1_, per_2_, per_3_, ..., per_*n*_} of all the pedestrians and set the number of initialization guides *K*=1, that is, it is calculated from the time when only one guide is sent.
Step 2: Calculate the initial Gaussian component parameters of the guide via the mean method, as shown in
  (3) $$\begin{array}{c} \mu_{k}^{\left(0\right)}=\frac{\sum_{j=1}^{n_{k}}{per}_{j}}{n_{k}}\text{,} \end{array}$$
  (4) $$\begin{array}{c} {\left(\sigma_{k}^{\left(0\right)}\right)}^{2}=\frac{\sum_{j=1}^{n_{k}}{\left({per}_{j}-\mu_{k}^{\left(0\right)}\right)}^{2}}{n_{k}}\text{,} \end{array}$$
  (5) $$\begin{array}{c} \omega_{k}^{\left(0\right)}=\frac{n_{k}}{n}\text{,} \end{array}$$
where *n*_*k*_ is the initial sampling quantity of the *k*-th guide *g*_*k*_.
Step 3: Start the inner loop iteration and set *i*=1.
Step 4: Execute Step *E* to calculate the posterior probability *γ*_*k*_^(*i*)^(per_*j*_) of each pedestrian belonging to guide *g*_*k*_, as shown in
  (6) $$\begin{array}{c} \gamma_{k}^{\left(i\right)}\left(x_{j}\right)=\frac{\omega_{k}^{\left(i\right)}p_{k}\left({per}_{j}\left|\Theta^{\left(i\right)}\right.\right)}{\sum_{k=1}^{K}\omega_{k}\cdot p_{k}\left({per}_{j}\left|\Theta^{\left(i\right)}\right.\right)}\text{,} \end{array}$$
where Θ^(*i*)^ is the parameter set of the current *i*-th iteration.
Step 5: Execute step *M* to calculate the parameter set of each guide via
  (7) $$\begin{array}{c} \mu_{k}^{\left(i+1\right)}=\frac{\sum_{j=1}^{n}\gamma_{k}^{\left(i\right)}\left({per}_{j}\right)\cdot {per}_{j}}{\sum_{j=1}^{n}\gamma_{k}^{\left(i\right)}\left({per}_{j}\right)}\text{,} \end{array}$$
  (8) $$\begin{array}{c} {\left(\sigma_{k}^{\left(i+1\right)}\right)}^{2}=\frac{\sum_{j=1}^{n}\gamma_{k}^{\left(i\right)}\left({per}_{j}\right)\cdot \left({per}_{j}-\mu_{k}^{\left(i+1\right)}\right)\cdot {\left({per}_{j}-\mu_{k}^{\left(i+1\right)}\right)}^{T}}{\sum_{j=1}^{n}\gamma_{k}^{\left(i\right)}\left({per}_{j}\right)}\text{,} \end{array}$$
  (9) $$\begin{array}{c} \omega_{k}^{\left(i+1\right)}=\frac{1}{n}\cdot \gamma_{k}^{\left(i\right)}\left({per}_{j}\right)\text{.} \end{array}$$
Step 6: If the convergence conditions are met, stop the inner loop iteration, that is, the best cluster has been identified, and the setting scheme when the number of guides is *K* is obtained; otherwise, execute the next iteration. The specific judgment statements are presented as follows:
  if ‖Θ^(i+1)^ − Θ^(i)^‖ < *ε*, then proceed to Step 7;
  else, *i*=*i*+1, skip to Step 4.
Step 7: Calculate the Bayesian information standard with the current number of guides as *K*: $BIC=k\;\ln\left(n\right)-2l\left(\hat{L}\right)$.
Step 8: *K*=*K*+1; continue to explore the next model.
Step 9: Judge whether the number of guides *K* reaches the maximum number of guides *K*_max_. If *K* ≥ *K*_max_, execute Step 10; otherwise, repeat Steps 2 to 7.
Step 10: When the algorithm stops, use the model corresponding to the lowest *BIC* value as the optimal model. The parameter set *θ*_*k*_^*∗*^ is the setting parameter of the optimal guide, *K* is the number of optimal guides, and *μ*_*k*_^*∗*^ is the optimal initial position of each guide in the optimal model.
Step 11: The algorithm ends.

## 3. Pedestrian Decision System Based on Fuzzy Inference

In the current crowd evacuation model with guides, it is generally assumed that pedestrians fully follow the guide assignment strategy [28]. However, pedestrians do not entirely obey the assigned guides in an actual evacuation due to individual differences in cognition and decision-making. Therefore, a two-layer fuzzy inference system for pedestrian decisions is established to reflect pedestrians' independent choice behavior when facing multiple guides at the site.

### 3.1. Influencing Factors of Pedestrian Decision Behavior

Pedestrian decision behavior is a complex system, especially during an emergency evacuation. Both emotion and a sudden change in the environment will affect people's perception system, resulting in people's fuzzy and imprecise judgment and choice of an external environment [29]. Cao et al. [30] proposed 20 elements to construct 216 fuzzy rules to describe the complexity of pedestrian movement. The model involves many factors, and coupling occurs between two factors. Yang et al. [31] proposed that the selection mechanism of pedestrians for guides mainly pertains to two aspects: the distance from guides and the density of people around guides. However, if the exit is within the visual range of pedestrians and the exit distance is small, individuals will independently evacuate from the reality of crowd evacuation [32, 33]. Therefore, the exit information is one of the influencing factors determining whether pedestrians choose to follow the guide.

In this paper, two influencing factors of pedestrians on the decision-making of guides are defined: the guide attraction factor *A*_1_ and the exit attraction factor *A*_2_. Moreover, these two factors are affected by the distance between the guide or exit and the pedestrian, the crowd density of the guide or exit, and whether the guide or exit is within the visual range of the pedestrian. Therefore, a multifactor fuzzy inference system is constructed. The system can reflect the uncertainty and variability of pedestrians when they perceive different information and simulate the decision behavior of pedestrians to guide.

### 3.2. Fuzzy Inference System for Pedestrian Decision

The fuzzy inference system of pedestrian decisions mainly uses fuzzy theory to reflect the inference process of pedestrians. The fuzzy inference process mainly includes four steps: fuzzification, fuzzy rules, the fuzzy inference process, and defuzzification. The purpose of fuzzification is to determine the domain of the input variables, fuzzy language set, and membership function and to implement the conversion of accurate numbers to fuzzy numbers to carry out logical operations with other fuzzy sets [29]. To make the pedestrian decision inference system more applicable, first, the numerical data input by the system are normalized. Second, the data are fuzzified and transformed into fuzzy language. Third, the fuzzy rules between the input variables and the output variables are defined. Last, defuzzification is performed to obtain the accurate value of the pedestrian guide selection probability.

The first layer of the constructed two-layer fuzzy inference system is the guide attraction decision system *R*_1_ and the exit attraction decision system *R*_2_. The three input variables of *R*_1_ are the orientation angle *αg* of the guide, the relative distance dis*g* from the guide, and the crowd density *ρg* around the guide. The output variable of *R*_1_ is the attraction factor *A*_1_ of the guide. The three inputs of *R*_2_ are the angle *αe* between the pedestrian and the exit line of sight, the relative distance dis*e* between the pedestrian and the exit, and the crowd density *ρe* around the exit. The output variable of *R*_2_ is the exit attraction factor *A*_2_. The second layer is the pedestrian decision system *R*_0_ for guidance. The input variables of *R*_0_ are the outputs *A*_1_ and *A*_2_ of the first layer, and the output variable is the selection probability *F*_*k*_ of the individual to the guide.

In the two-layer fuzzy inference system, the orientation, distance, and pedestrian density around the guides or exits are three important factors, but they all lack clear standards and belong to fuzzy concepts. Constructing a membership function is one of the keys to the fuzzy system. At present, the methods of establishing the membership function of most fuzzy systems are based on experience or experiment. Everyone has a different understanding of the same fuzzy concept, so the determination of membership function is subjective. Gaussian, bell, and triangular membership functions are the three commonly used membership functions in fuzzy systems, which are most close to the distribution of actual observation data. In recent years, scholars have started to describe pedestrian evacuation variables with fuzzy mathematics to make the crowd evacuation simulation model more practical. Yang et al. [15] selected Gaussian and bell membership functions as the distance variable from the follower to the guide and the pedestrian density variable around the guide, and selected the triangular function as the guide selection probability variable. Cao et al. [30] proposed the Gaussian and bell membership functions as the pedestrian behavior variables, and the triangular function as the decision variable of pedestrian movement, and collected the motion data of 2484 pedestrians to calibrate the membership functions. Based on the existing studies, Gaussian and bell membership functions are selected as distance and density variables, and the triangular function is selected as the output variable of each layer of the fuzzy inference system. Li et al. [34] proposed that the fuzzy visual range of pedestrians is a 90-degree fan. By using the method of selecting and assigning the membership function, it is analyzed that the visual field of pedestrians in a certain direction follows the ridge distribution. Liu et al. [35] defined three different membership functions through the tripartite method, aiming to clarify the random interval of the analysis object. On the basis of above, this paper considered that although the orientations (Front, Besides, and Back) of the guides or exits are affected by the visual range of pedestrians, it is easier to make a deviation in the judgment of the three orientations. Therefore, Gaussian and bell membership functions are used to represent the three orientation angle variables of the guides and exits, and each interval represents the correlation between the orientation angles and the three orientations according to the concept of the tripartite method. The fuzzy inference process of each decision system will be described.

#### 3.2.1. Guide Attraction Decision System *R*_1_

The input variable *αg* represents the variable after the normalization of the orientation angle of the guide, as shown in

(10) $$\begin{array}{c} \alpha g=\frac{1}{\left[1+\exp\;\left(-\;\left(\left|\alpha_{j}-{angle}_{j}^{k}\right|/\pi \right)\right)\right]}\text{,} \end{array}$$

where *α*_*j*_ is the angle between the direction vector of individual *j* and the positive direction of the *X* axis, and its direction vector calculation formula is $\alpha_{j}^{t}=\arctan\;\left[\left({\tilde{y}}_{j}^{t}-y_{j}^{t-1}\right)/\left({\tilde{x}}_{j}^{t}-x_{j}^{t-1}\right)\right]$, where (*x*_*j*_^*t*−1^, *y*_*j*_^*t*−1^) is the position coordinate of individual *j* at time *t* − 1, $\left({\tilde{x}}_{j}^{t},{\tilde{y}}_{j}^{t}\right)$ is the position coordinate of individual *j* at time *t*, and angle_*j*_^*k*^ is the angle between the position of individual *j* and that of guide *g*_*k*_.

**Figure 1:**
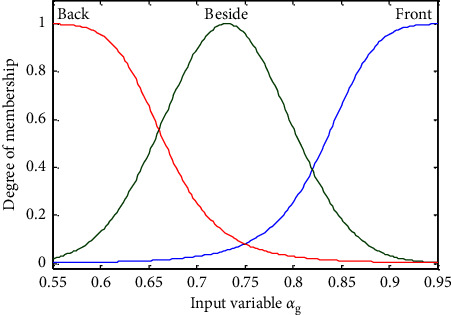
Membership function of orientation angle *αg* of the guide.

After normalization, the value range of *αg* is [0,1]. When the guide is directly in front of the individual, *αg*=1, and when the guide is directly behind the individual, *αg*=0. The linguistic value of *αg* is {the guide is in the front (Front), the guide is on the side (Beside), and the guide is in the back (Back)}. Gaussian and bell membership functions are employed, as shown in Figure 1.

The input variable dis*g* represents the variable after normalization of the distance between pedestrian per_*j*_ and guide *g*_*k*_, and its calculation formula is dis*g*=distance(per_*j*_, *g*_*k*_)/*R*_visual_, where distance(per_*j*_, *g*_*k*_) is the Euclidean distance between guide *g*_*k*_ and pedestrian per_*j*_, *R*_visual_ is the visible range radius of pedestrians in a crisis scenario, and the range of dis*g* after normalization is [0,1].

The linguistic value of dis*g* is {the distance is near (Near), the distance is middle (Middle), and the distance is far (Far)}, and the Gaussian and bell membership functions are also applied.

The input variable *ρg* represents the variable after normalizing the number of people around guide *g*_*k*_, and its calculation formula is *ρg*=1/[1+exp (−(*N*_*g*_/*N*_*in*_)/*K*)], where *N*_*g*_ is the number of people around guide *g*_*k*_, *N*_*in*_ is the number of people currently staying indoors, and *K* is the number of guides at the site.

The linguistic value of *ρg* is {the density is high (High), the density is medium (Medium), and the density is low (Low)}, and the Gaussian and Bell membership functions are similarly applied.

**Figure 2:**
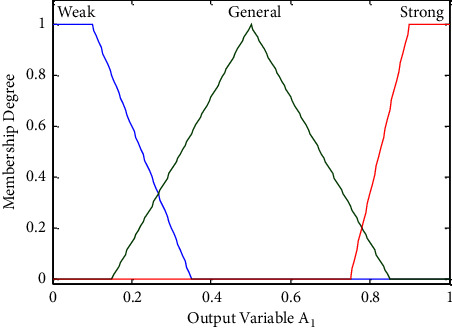
Membership degree of attraction factor *A*_1_ of the guide.

As the output variable, the linguistic value of *A*_1_ is {the attraction is strong (Strong), the attraction is general (General), and the attraction is weak (Weak)}, and the triangular and trapezoidal membership functions are utilized, as shown in Figure 2.

The fuzzy inference system *R*_1_ for calculating the guide attraction factor *A*_1_ is expressed as follows:

(11) $$\begin{array}{c} A_{1}=R_{1}\left(disg,\rho g,\alpha g\right)\text{.} \end{array}$$

The input variables dis*g*, *ρg*, and *αg* have three linguistic values, so there are 27 *if* − then inference rules. The basic principle of the inference rule setting is that if the distance is near, the density is low; if the guide is in the front, the attraction of the guide is strong; if the distance is far, the density is high; and if the guide is in the back, the attraction of the guide is weak. The partial rules of the guide attraction decision system *R*_1_ are listed as follows:

  If disg is near, *ρ*g is low, and *α*g is in the front, then A_1_ is strong
  If disg is near, *ρ*g is low, and *α*g is on the side, then A_1_ is strong
  If disg is near, *ρ*g is low, and *α*g is in the back, then A_1_ is weak
  …
  If disg is far, *ρ*g is high, and *α*g is in the back, then A_1_ is weak

#### 3.2.2. Exit Attraction Decision System *R*_2_

As input variables, the normalization process, fuzzy linguistic value, and membership function of *αe*, di s*e*, and *ρe* are consistent with the definition of the input variables of *R*_1_. As the output variable, the linguistic value of *A*_2_ is {the attraction is strong (Strong), the attraction is general (General), and the attraction is weak (Weak)}, using the same triangular and trapezoidal membership functions as *A*_1_.

Next, the fuzzy inference system *R*_2_ for calculating the exit attraction factor *A*_2_ is expressed as follows:

(12) $$\begin{array}{c} A_{2}=R_{2}\left(dise,\rho e,\alpha e\right)\text{.} \end{array}$$

The input variables dis*e*, *ρe*, and *αe* have three linguistic values, so there are 27 *if* − then inference rules. The basic principle of the inference rule setting is that if the distance is near, the density is low; if the exit is in the front, the attraction of the exit is strong; if the distance is far, the density is high; and if the exit is in the back, the attraction of the exit is weak.

#### 3.2.3. Pedestrian Decision System *R*_0_ for the Guide

**Figure 3:**
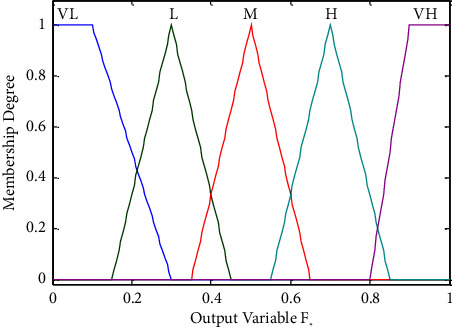
Membership degree of *F*_*k*_.

As input variables, the values of *A*_1_ and *A*_2_ can be calculated by the fuzzy inference systems *R*_1_ and *R*_2_. As an output variable, *F*_*k*_ represents the probability *F*_*k*_ of pedestrian selection guide *g*_*k*_, and the value range is [0,1]. The linguistic value of *F*_*k*_ is {the selected probability is very high (VH), the selected probability is high (H), the selected probability is medium (M), the selected probability is low (L), and the selected probability is very low (VL)} and the triangular and trapezoidal membership functions are utilized, as shown in Figure 3.

Next, the fuzzy inference system *R*_0_ for calculating the probability *F*_*k*_ of selection guide *g*_*k*_ is expressed as follows:

(13) $$\begin{array}{c} F_{k}=R_{0}\left(A_{1},A_{2}\right)\text{.} \end{array}$$

The input variables *A*_1_ and *A*_2_ have three linguistic values, so there are 9 *if* − then inference rules. The basic principle of the inference rule setting is that if the attraction of the guide is strong, the selected probability of the guide is high; if the attraction of the exit is strong, the selected probability of the guide is low.

In this model, the centroid method is used to defuzzify, and the output of the *R*_0_ system is transformed into an accurate probability value *F*_*k*_^*∗*^; that is, the selection probability of pedestrians to guide *g*_*k*_ is obtained.

### 3.3. Social Force Model of Crowd Evacuation with Guides

In the process of evacuation, the guides and pedestrians form a group effect, and the social force model (SFM) can effectively simulate the characteristics of a group movement. Therefore, the exit selection mechanism of guides and the movement mechanism of pedestrians with improved social force are constructed in the model based on the guide setting scheme and pedestrian decision inference system.

### 3.4. Exit Selection Mechanism of Guide

In the classical social force model, the individual force includes the self-driving force $\overset{⟶}{f_{j}^{a}}\left(t\right)$, the force between two individuals $\overset{⟶}{f_{ji}}\left(t\right)$, and the force between the individual and the wall or obstacle $\overset{⟶}{f_{jW}}\left(t\right)$. The calculation of the self-driving force $\overset{⟶}{f_{j}^{a}}\left(t\right)$ is shown in

(14) $$\begin{array}{c} \overset{⟶}{f_{j}^{a}}\left(t\right)=m_{j}\frac{\overset{⟶}{v_{j}^{0}}\left(t\right)\cdot \overset{⟶}{e_{j}^{0}}\left(t\right)-\overset{⟶}{v_{j}}\left(t\right)}{\tau_{j}}\text{,} \end{array}$$

where *m*_*j*_ is the mass of individual *j*; $\overset{⟶}{v_{j}^{0}}\left(t\right)$ and $\overset{⟶}{e_{j}^{0}}\left(t\right)$ are the expected speed and expected direction, respectively, of individual *j* during evacuation; *τ*_*j*_ is the time interval for individual *j* to adjust its own speed; and $\overset{⟶}{v_{j}}\left(t\right)$ is the adjusted actual speed.

$\overset{⟶}{f_{ji}}\left(t\right)$ is the interaction force between individual *j* and individual *i*, which is composed of repulsive force $\overset{⟶}{f_{ji}^{R}}\left(t\right)$, body resistance force $\overset{⟶}{f_{ji}^{B}}\left(t\right)$, and sliding friction force $\overset{⟶}{f_{ji}^{S}}\left(t\right)$. Therefore, the calculation of $\overset{⟶}{f_{ji}}\left(t\right)$ is shown in

(15) $$\begin{array}{c} \left\{\begin{array}{c} \overset{⟶}{f_{ji}}\left(t\right)=\overset{⟶}{f_{ji}^{R}}\left(t\right)+\overset{⟶}{f_{ji}^{B}}\left(t\right)+\overset{⟶}{f_{ji}^{S}}\left(t\right)\\ =A_{j}\exp\left[\frac{\left({rad}_{ji}-d_{j}\right)}{B_{j}}\right]\cdot \overset{⟶}{n_{ji}}+k\cdot g\left({rad}_{ji}-d_{ji}\right)\cdot \overset{⟶}{n_{ji}}+\kappa \cdot g\left({rad}_{ji}-d_{ji}\right)\cdot \Delta v_{ij}\left(t\right)\cdot \overset{⟶}{t_{ji}}\text{,}\\ g\left({rad}_{ji}-d_{ji}\right)=\left\{\begin{array}{cc} 0\text{,} & d_{ji}\geq {rad}_{ji}\text{,}\\ {rad}_{ji}-d_{ji,} & d_{ji}<{rad}_{ji}\text{,} \end{array}\right. \end{array}\right. \end{array}$$

where *A*_*j*_, *B*_*j*_, *k*, and *κ* are constants; $\overset{⟶}{n_{ji}}=\left(\overset{⟶}{n_{ji}^{1}},\overset{⟶}{n_{ji}^{2}}\right)=\left(r_{j}\left(t\right)-r_{i}\left(t\right)\right)/d_{ji}$ is the normalized vector from individual *j* to individual *i*; *d*_*ji*_=‖*r*_*j*_(*t*) − *r*_*i*_(*t*)‖ is the distance between the mass centers of individual *j* and individual *i*; rad_*ji*_=(rad_*j*_+rad_*i*_) is the sum of the body radii of individual *j* and individual *i*; and $\overset{⟶}{t_{ji}}=\left(-n_{ij}^{2},n_{ij}^{1}\right)$ indicates the tangential direction, and $\Delta \overset{⟶}{v_{ij}}\left(t\right)=\left(\overset{⟶}{v_{i}}\left(t\right)-\overset{⟶}{v_{j}}\left(t\right)\right)\cdot \overset{⟶}{t_{ji}}$ indicates the tangent speed difference.

Individuals *j* are affected by the surrounding environment during movement. If these individuals encounter obstacles or walls of enclosed spaces, they usually choose to avoid them in advance. When avoiding obstacles or walls *W*, the corresponding interaction force $\overset{⟶}{f_{jW}}\left(t\right)$ is shown in

(16) $$\begin{array}{c} \overset{⟶}{f_{jW}}\left(t\right)=\overset{⟶}{f_{jW}^{R}}\left(t\right)+\overset{⟶}{f_{jW}^{B}}\left(t\right)+\overset{⟶}{f_{jW}^{S}}\left(t\right)\\ =A_{j}\exp\;\left[\frac{\left({rad}_{j}-d_{jW}\right)}{B_{j}}\right]\cdot \overset{⟶}{n_{jW}}+k\cdot g\left({rad}_{j}-d_{jW}\right)\cdot \overset{⟶}{n_{jW}}-\kappa \cdot g\left({rad}_{j}-d_{jW}\right)\cdot \left(v_{j}\cdot \overset{⟶}{t_{jW}}\right)\cdot \overset{⟶}{t_{jW}}\text{,} \end{array}$$

where $\overset{⟶}{n_{jW}}$ is the direction perpendicular to the wall or obstacle, and $\overset{⟶}{t_{jW}}$ is the tangent direction with the wall or obstacle.

The guide is usually very familiar with the environment at the site and the exit location, and is not affected by environmental factors such as the field radius. During the process of evacuation, the guide determines the exit with less evacuation time as the target exit by estimating the evacuation time of each exit, which can be applied as the forward direction of the self-driving force $\overset{⟶}{f_{j}^{a}}\left(t\right)$ in the social force model. Therefore, the exit selection mechanism of the guide is constructed as follows.

It is supposed that pre − time_i_ is the estimated evacuation time from guide *g*_*k*_ to exit *E*_*i*_, which is composed of the estimated walking time *W* − time_*i*_ from guide *g*_*k*_ to exit *E*_*i*_ and the queuing waiting time *Q* − time_*i*_ at exit *E*_*i*_. The exit with the smallest estimated evacuation time is the target exit *E*_*s*_^*∗*^. The minimum estimated evacuation time is shown in

(17) $$\begin{array}{c} pre-{time}_{s}^{*}=\min\;\left(pre-{time}_{i}\right),E_{i}\in exitE\\ =\min\;\left(W-{time}_{i}+Q-{time}_{i}\right)\text{,} \end{array}$$

where exit*E* is the set of all exits at the site, *E*_*i*_ is the *i*-th exit at the site, i.e., *E*_*i*_ ∈ exit*E*, pre − time_*i*_ is the estimated evacuation time from guide *g*_*k*_ to exit *E*_*i*_, and *E*_*s*_^*∗*^ is the optimal exit.

The estimated walking time *W* − time_*i*_ to the exit is the ratio of the distance between guide *g*_*k*_ and exit *E*_*i*_ to the walking speed of guide *g*_*k*_, as shown in

(18) $$\begin{array}{c} W-{time}_{i}=\frac{\left\|r_{g_{k}}-r_{E_{i}}\right\|}{v_{g_{k}}^{\left(t\right)}}\text{.} \end{array}$$

The queue waiting time *Q* − time_*i*_ in the exit area is the ratio of the number of people leaving from exit *E*_*i*_ to the traffic capacity of exit *E*_*i*_, as shown in

(19) $$\begin{array}{c} Q-{time}_{i}=\frac{dens\left(E_{i}\right)}{capacity\left(E_{i}\right)}\text{,} \end{array}$$

where de ns(*E*_*i*_) is the sum of the number of people who choose exit *E*_*i*_ that can be observed by guide *g*_*k*_, and capacity(*E*_*i*_) is the traffic capacity of exit *E*_*i*_, that is, the number of people who can pass per unit time.

Thus, the speed of guide *g*_*k*_ is shown in

(20) $$\begin{array}{c} M_{g_{k}}\frac{dv_{g_{k}}\left(t\right)}{dt}=M_{g_{k}}\frac{\overset{⟶}{v_{g_{k}}^{0}}\left(t\right)\cdot \overset{⟶}{e_{g_{k}}^{0}}\left(t\right)-v_{g_{k}}\left(t-\tau \right)}{\tau }+\overset{⟶}{f_{g_{k}i}}\left(t\right)+\overset{⟶}{f_{g_{k}W}}\left(t\right)\text{,} \end{array}$$

where the desired forward direction $\overset{⟶}{e_{g_{k}}^{0}}$ is determined by the optimal exit *E*_*s*_^*∗*^, as shown in

(21) $$\begin{array}{c} \overset{⟶}{e_{g_{k}}^{0}}=\frac{\left(r_{E_{s}^{*}}-r_{g_{k}}\right)}{\left\|r_{E_{s}^{*}}-r_{g_{k}}\right\|}\text{.} \end{array}$$

### 3.5. Movement Mechanism of the Pedestrians

During the process of evacuation, if an exit is within the visual range of pedestrians, then the pedestrians will independently evacuate; otherwise, the pedestrians will choose to follow the guide to evacuate. Therefore, the movement mechanism of the pedestrians is formulated, and the driving force function of the evacuating individuals is redefined, as shown in

(22) $$\begin{array}{c} M_{j}\frac{dv_{j}\left(t\right)}{dt}=\overset{⟶}{f_{j}^{a-new}}\left(t\right)+\overset{⟶}{f_{ji}}\left(t\right)+\overset{⟶}{f_{jW}}\left(t\right)\text{,} \end{array}$$

where $\overset{⟶}{f_{j}^{a-new}}\left(t\right)$ is the evacuation driving force of pedestrian *j*, as shown in

(23) $$\begin{array}{c} \overset{⟶}{f_{j}^{a-new}}\left(t\right)=de_{j}\left(t\right)\cdot \overset{⟶}{f_{j}^{a}}\left(t\right)+\left(1-de_{j}\left(t\right)\right)\cdot \overset{⟶}{f_{j}^{g}}\left(t\right)\text{,} \end{array}$$

where *de*_*j*_(*t*) is a Boolean variable. When the distance between pedestrian *j* and the exit is less than or equal to the visible distance *d*_min, the variable is 1; that is, the forward driving force of pedestrian *j* is attracted by the exit. When pedestrian *j* cannot see any exit, the variable is 0; that is, pedestrian *j* completely follows the guide. Therefore, *de*_*j*_(*t*) is expressed as

(24) $$\begin{array}{c} de_{j}\left(t\right)=Boolean\left(\left\|r_{E_{k}}-r_{j}\right\|<=d\_min\right)\text{,} \end{array}$$

where Boolean(*·*) is the Boolean decision function.

$\overset{⟶}{f_{j}^{g}}\left(t\right)$ in equation (23) is calculated as follows:

(25) $$\begin{array}{c} \overset{⟶}{f_{j}^{g}}\left(t\right)=M_{j}\frac{\overset{⟶}{v_{j}^{0}}\left(t\right)\cdot \overset{⟶}{ge_{j}^{0}}\left(t\right)-v_{j}\left(t-\tau_{j}\right)}{\tau_{j}}\text{,} \end{array}$$

where $\overset{⟶}{ge_{j}^{0}}$ is the desired direction of pedestrian *j* aimed at the position of the guide, which drives pedestrian *j* to approach guide *g*_*k*_, as shown in

(26) $$\begin{array}{c} \overset{⟶}{ge_{j}^{0}}=\frac{\left(r_{g_{k}}-r_{j}\right)}{\left\|r_{g_{k}}-r_{j}\right\|}\text{.} \end{array}$$

### 3.6. Simulation Algorithm Flow of the Model

In the previous section, a crowd evacuation model with guides is constructed. First, the number and optimal locations of the guides are determined according to the crowd distribution at the site. Next, the pedestrian makes an independent decision to choose the guide to follow. On this basis, the exit selection mechanism of the guides and the movement mechanism of the pedestrians are defined. The algorithmic steps of the crowd evacuation model with guides under a limited vision scenario are listed as follows:

  Step 1: Scene, crowd, and model parameters are initialized.
  Step 2: The Gaussian fuzzy clustering method is used to determine the number K^*∗*^ of guides and the optimal position of the guides according to the initial position of the crowd.
  Step 3: The total evacuation time is set to t = 0 s when the evacuation starts.
  Step 4: The pedestrian determines the guide with the highest probability as the following target according to an independent decision, that is, F_k_^*∗*^=max {F_k_, k ∈ K}. If F_k_^*∗*^=∅, the pedestrian does not choose to follow the guide.
  Step 5: The guide selects the desired exit according to equations (17)–(19), modifies the self-driving force, and moves according to equations (20)-(21). The pedestrian following the guide modifies the self-driving force and moves according to equations (22)–(25). Pedestrians who do not choose to follow the guide continue the original forward strategy.
  Step 6: The number of pedestrians leaving each exit is recorded. If all pedestrians are evacuated, we jump to Step 8; otherwise, we proceed to Step 7.
  Step 7: Whether the total evacuation time T reaches the maximum time is determined. If the maximum execution time has been reached, we proceed to Step 8; otherwise, we jump to Step 4.
  Step 8: The algorithm ends. The number of people at each exit and the total evacuation time are calculated.

## 4. Experimental Simulation

In this paper, the evacuation time of the guide setting method of the improved model and the existing methods in several scenes is analyzed and compared through simulation. In addition, the evacuation process of several guide settings under different population distributions is analyzed. The effectiveness and superiority of the improved model are verified by the following experiments.

### 4.1. Experiment 1

To analyze the effectiveness of the guide setting method proposed in this model, four typical room types are selected as the simulation experiment scene. The scenes are based on a simulated square room with a size of 20m × 20m. The width of the exit is 1.5 m. The exit positions of each room differ, and the environmental parameters of the scenes are described as follows:

1. Scene 1: A relatively closed space with only a single exit is simulated, and the exit is located in the middle of a wall
2. Scene 2: A classroom or conference room with double exits on the same side is simulated, and the two exits are located far away on a single wall
3. Scene 3: A public place with two emergency exits is simulated, and the exits are located in the middle of the two opposite walls
4. Scene 4: A cinema or theatre with three exits is simulated, and the exits are located in the middle of the three walls

**Figure 4:**
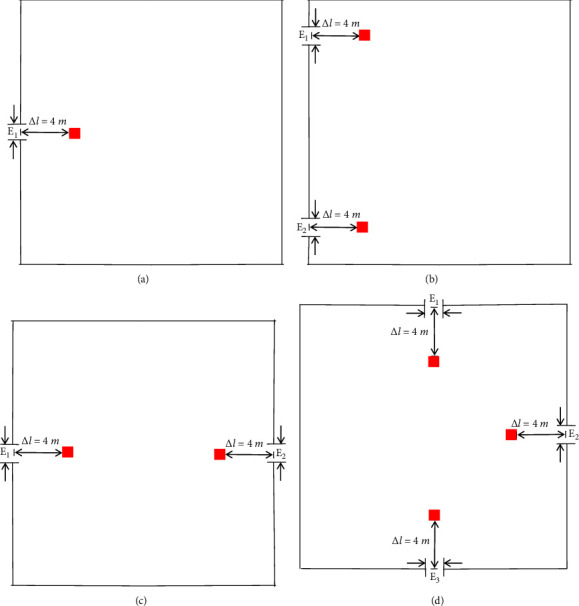
Guide position of the near-exit method in different scenes. (a) Scene 1. (b) Scene 2. (c) Scene 3. (d) Scene 4.

**Figure 5:**
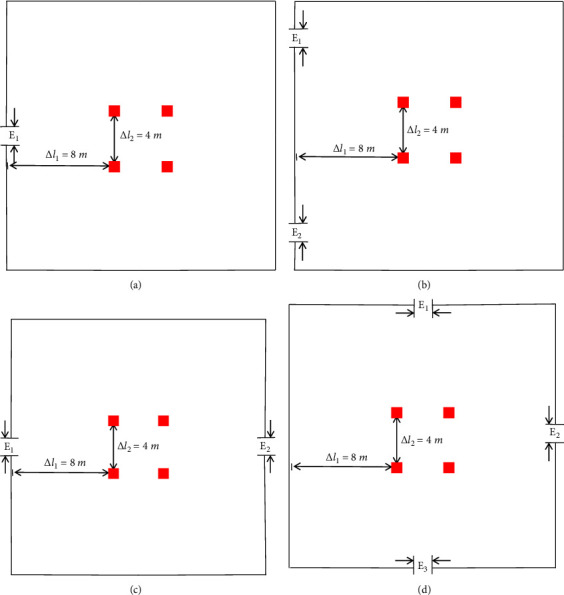
Guide position of the center method in different scenes. (a) Scene 1. (b) Scene 2. (c) Scene 3. (d) Scene 4.

For the setting method of the guide, some researchers proposed setting the guide at a fixed position in front of the exit [12], while other researchers proposed setting the guide at the center of the room [13]. In this paper, the improved social force model proposes that the initial position of the guide is related to the crowd distribution at the site. The Gaussian fuzzy clustering method is selected to determine the optimal initial position of the guide according to the crowd position coordinates. To facilitate analysis and comparison, the three methods are named the near-exit method [12], the center method [13], and the GMM method. The positions of the guides of the near-exit method and the center method in the four scenes are shown in Figures 4 and 5, respectively.

**Figure 6:**
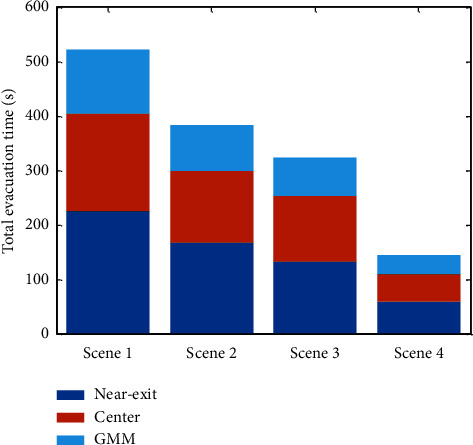
The evacuation time of the three methods in different scenes.

In the simulation experiment, 200 people were randomly distributed in the room. The visibility is set to 4 m, that is, *d*_ min =4m. The sampling time of the social force evacuation model is set to 0.2 s. The simulation program is executed 100 times in each case, and the average values are calculated. The total evacuation time of the three methods in the four scenes is shown in Figure 6.

As depicted in Figure 6, when the initial number of pedestrians is the same, the evacuation time of the GMM method in the four scenes is less than that of the other two methods as the initial position of the guide that is 4 m from the exit in the near-exit method. When the guide leaves the exit, some pedestrians behind the guide cannot see the exit position, so they walk in a random manner. The figure shows that the total evacuation time of the center method is better than that of the near-exit method as when the guide leads the pedestrian to move in the center method, the movement streamline angle of the pedestrian is changed, so the problems caused by the near-exit method are avoided. However, the center method, in which the guide is concentrated in the central area of the site, also has some shortcomings. Pedestrians who choose the guide need to gather in the central area of the site and then move to the exit. Pedestrians may increase their travel distance, resulting in an increase in evacuation time. In the GMM method, the guide is clustered according to the population distribution, and the clustering objective function is to minimize the difference in the distance to each cluster center, which not only improves the situation that some people have no guidance due to the guide leaving too fast but also avoids the excessive travel distance due to the tendency of people to move to the guide. The GMM method is more conducive to rapid crowd evacuation than the near-exit and center methods.

### 4.2. Experiment 2

The location of an indoor crowd is an important factor of the effectiveness of a guidance strategy. In this section, selecting Scene 4 as an example, the evacuation process of the three guide setting methods in the situation of uniform population distribution with visibility *d*_min=4m is analyzed.

**Figure 7:**
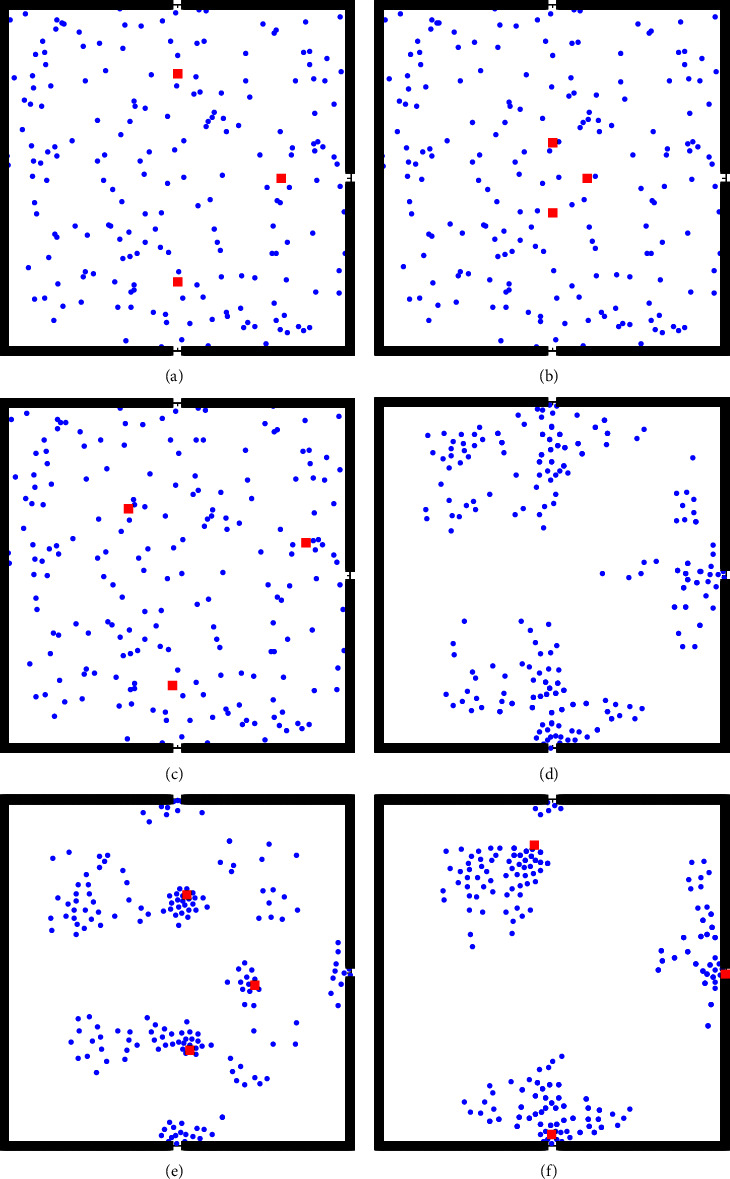
Evacuation state diagram of the uniform distribution of the initial population. (a) Near-exit method (*t* = 0 s). (b) Center method (*t* = 0 s). (c) GMM method (*t* = 0 s). (d) Near-exit method (*t* = 5 s). (e) Center method (*t* = 5 s). (f) GMM method (*t* = 5 s).

The initial positions of the uniformly distributed pedestrians are randomly generated. The initial positions (*t* = 0 s) of the different methods are shown in Figures 7(a)–7(c). The evacuation status after 5 s is shown in Figures 7(d)–7(f).  Figure 7 shows obvious differences in the evacuation status of the different methods. When *t* = 5 s, the guide of the near-exit method has left the site, and the original pedestrians can only independently evacuate. Pedestrians in the center method move toward the guide and form a gathering around the guide. However, when the pedestrian approaches the guide, the pedestrian moves in the opposite direction to the exit as the guide is in the center. The guide of the GMM method is closer to the exit than the guide of the center method, and the surrounding people form a gathering. In addition, pedestrians will not walk in the opposite direction for a long distance. When *t* = 5 s, the guide is still at the site and can continue to lead the crowd to evacuate.

**Figure 8:**
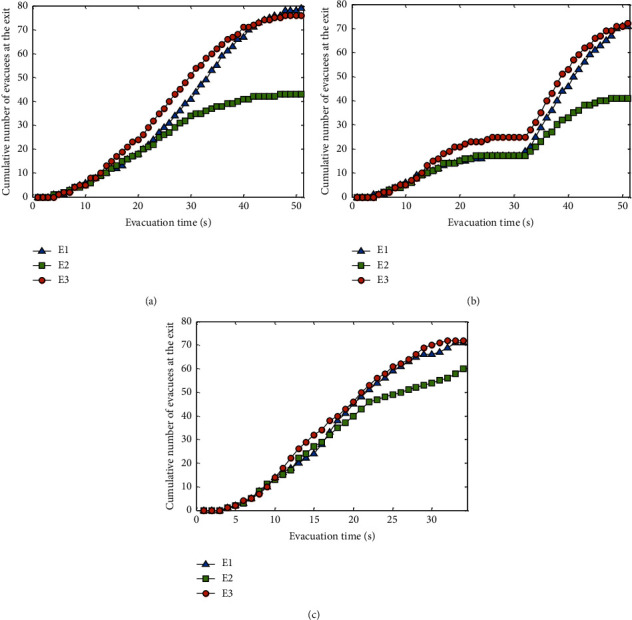
Cumulative number of evacuees at each exit with a uniformly distributed initial population. (a) Near-exit method. (b) Center method. (c) GMM method.

To analyze the effects of the three methods on the evacuation efficiency, *N*_*E*_*k*__(*t*) is defined to represent the cumulative number of people leaving from exit *E*_*k*_ from the beginning of the evacuation to time *t*, that is, *N*_*E*_*k*__(*t*)=*N*_*E*_*k*__(*t* − 1)+*n*_*E*_*k*__(*t*), where *n*_*E*_*k*__(*t*) is the number of people escaping from exit *E*_*k*_ at time *t*. The cumulative number of people evacuated, *N*_*E*_*k*__(*t*), by the three methods is shown in Figure 8.

As depicted in Figure 8, there is an imbalance in the number of evacuees among the exits in both the near-exit and center methods. However, the difference in *N*_*E*_*k*__(*t*) among the three exits of the GMM method is less than that of the other two methods, indicating that the GMM method is more conducive to the balance of the evacuation number at the exit. For example, in Figure 8(b), *N*_*E*_*k*__(*t*) of the three exits with the center method has not changed from *t* = 22 s to *t* = 32 s, indicating that no personnel has evacuated from the exit during this period. The reason is that the guide in the center method is in the central position, and the pedestrians gather around the guide, causing some pedestrians to move in the opposite direction away from the exit. These pedestrians are concentrated in the central area and then move to the exit, yielding the phenomenon that there was no evacuation at the exit for a certain period.

### 4.3. Experiment 3

**Figure 9:**
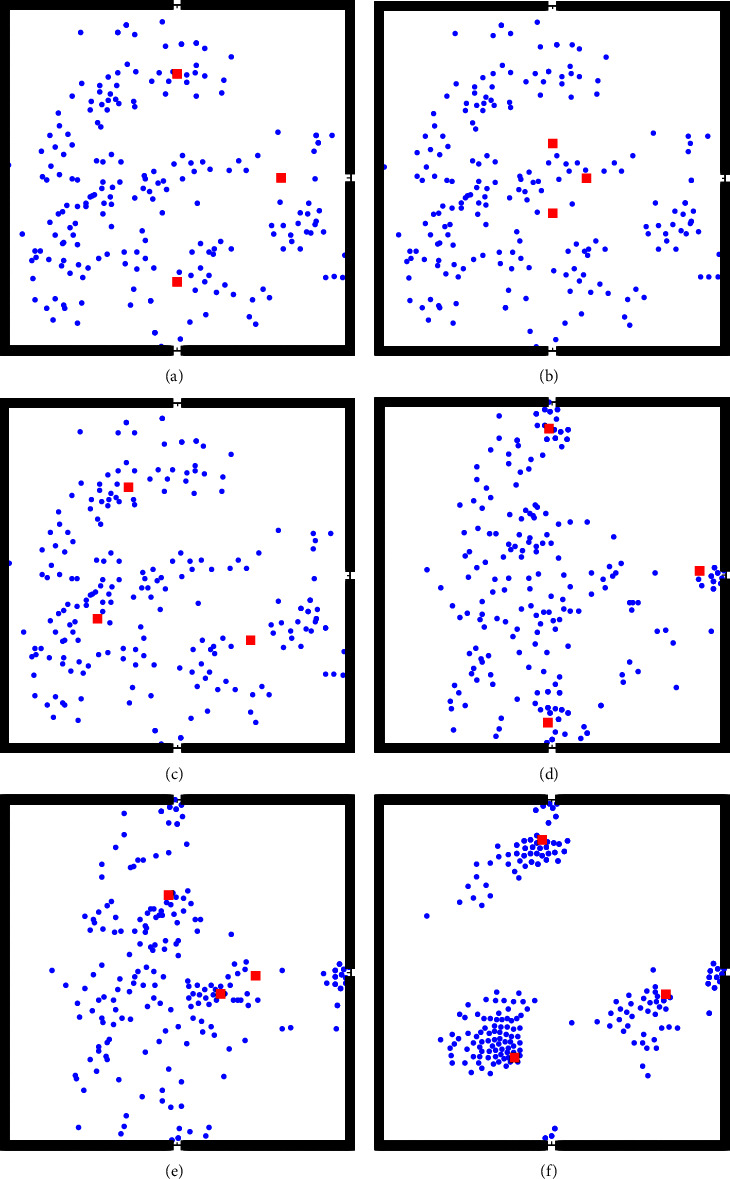
Evacuation state diagram of the nonuniform distribution of the initial population. (a) Near-exit method (*t* = 0 s). (b) Center method (*t* = 0 s). (c) GMM method (*t* = 0 s). (d) Near-exit method (*t* = 5 s). (e) Center method (*t* = 5 s). (f) GMM method (*t* = 5 s).

When the population is uniformly distributed at the site, the initial positions (*t* = 0 s) of the different methods are shown in Figures 9(a)–9(c), respectively; the evacuation status after 5 s is shown in Figures 9(d)–9(f), respectively.

As depicted in Figure 9(d), when *t* = 5 s, the crowd does not form obvious aggregations in the near-exit method. In this case, the pedestrians at the site will show a state of nonguided evacuation once the guide leaves the exit. Simultaneously, the corresponding crowd in the center method forms a gathering next to the guide, as shown in Figure 9(e). However, the two guides of this method choose the E2 exit to leave, so the pedestrians who choose these two guides will also leave through the E2 exit, resulting in an imbalance of exit utilization. As shown in Figure 9(f), the crowd gathered at the guide in the GMM method at *t* = 5 s, and the leading trend of the guides was obvious. The three guides chose three different exits.

**Figure 10:**
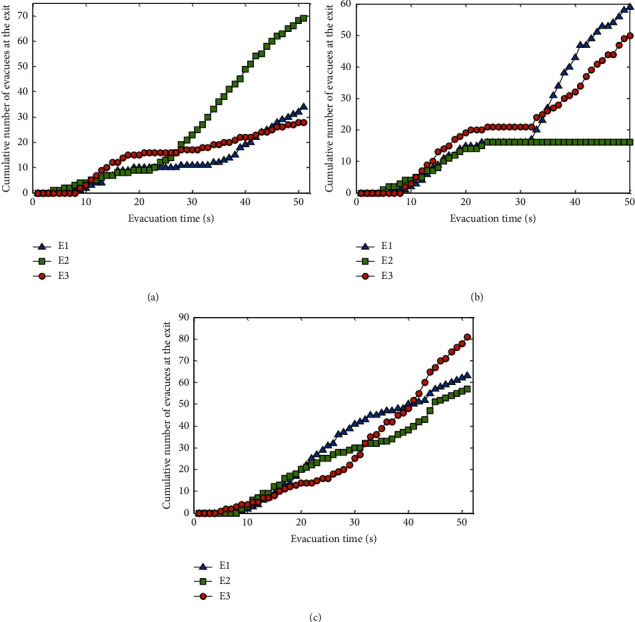
Cumulative number of evacuees at each exit with a nonuniformly distributed initial population. (a) Near-exit method. (b) Center method. (c) GMM method.

In the case of a nonuniform initial pedestrian distribution, the *N*_*E*_*k*__(*t*) of the three methods is shown in Figure 10. Although the exit imbalance also exists in the GMM method, the difference in the number of people at each exit is significantly smaller than that in the near-exit and center methods.

In summary, the GMM has more obvious advantages in evacuation time, crowd gathering state, equilibrium of export utilization, and crowd distribution compared with the near-exit and center methods.

## 5. Conclusions

In this paper, a multifactor fuzzy inference system integrating the guide attraction and exit attraction is designed, and an improved social force model that combines the guide setting scheme and pedestrian decision system is established. Through the simulation analysis of the model, the following conclusions are obtained:

1. Compared with the current methods, the near-exit method and center method, the GMM method proposed in this paper solves the problem that some pedestrians have no guidance due to the too fast departure of the guide and avoids the excessive travel distance due to the tendency to move toward the guide.
2. The number and location of the guides determined by the GMM method are suitable for different crowd location distributions, especially for nonuniformly distributed crowds. Compared with the near-exit and center methods, the improved method is more conducive to shortening the evacuation time and balancing the utilization rate of each exit.

The improved model is more consistent with an actual evacuation, which is helpful in providing a theoretical basis for guiding pedestrian evacuations in emergencies. But there are still some aspects to be improved in future research. The simulation results require the support of empirical data. Future empirical experiments will be carried out on the model to verify the significance of the conclusions. Moreover, a typical validation practice of the evacuation models is the generation of the corresponding fundamental diagram [36]. This robust qualitative and quantitative validation method is widely used in crowd evacuation modeling [37]. Therefore, we will use this fundamental diagram as the research object to further assess the efficiency of our method.

## Data Availability

The data used to support the findings of this study are available from the corresponding author upon request.
